# Effect of distraction length on the morphology of knee cartilage in a rat model of femoral distraction osteogenesis

**DOI:** 10.3389/fphys.2026.1779440

**Published:** 2026-03-16

**Authors:** Caifeng Wu, Yuanxin Chen, Yanshi Liu, Lian Tang, Xiaoheng Ding, Aihemaitijiang Yusufu, Kai Liu

**Affiliations:** 1 Department of Orthopaedics, The Affiliated Hospital of Southwest Medical University, Luzhou, Sichuan, China; 2 Department of Hand and Foot Microsurgery, Qingdao University Affiliated Hospital, Qingdao, Shandong, China; 3 Department of Trauma and Microreconstructive Surgery, The First Affiliated Hospital of Xinjiang Medical University, Urumqi, Xinjiang, China

**Keywords:** bone regeneration, bone remodeling, cartilage degeneration, distraction osteogenesis, osteoarthritis

## Abstract

**Objective:**

This study investigated the dose-dependent impact of femoral distraction length on knee joint integrity and characterized the molecular mechanisms driving cartilage degeneration in a rat distraction osteogenesis (DO) model.

**Methods:**

Thirty-six Sprague-Dawley rats underwent femoral DO and were randomly assigned to three groups: Control (5 mm), Group A (10 mm), and Group B (20 mm). Following a consolidation phase, knee joint morphology and subchondral bone microstructure were evaluated using digital radiography and micro-computed tomography. Histological assessment included H&E, Safranin O-Fast Green, and Masson’s trichrome staining. Furthermore, immunohistochemical analysis quantified the expression of catabolic (IL-1β, MMP-13, RANKL) and anabolic (COL-II, SOX9, OPG) biomarkers in the articular cartilage and subchondral bone.

**Results:**

Radiographic and histological findings demonstrated successful osseointegration and physiological tolerance in the Control and Group A. Conversely, Group B exhibited severe osteoarthritis-like pathology, including cartilage erosion, proteoglycan depletion and subchondral sclerosis. Quantitative analysis confirmed significantly elevated bone mineral density and bone volume fraction in the subchondral bone of Group B (*P* < 0.05). Molecularly, Group B showed significant upregulation of catabolic biomarkers (IL-1β, MMP-13 and RANKL) with concurrent downregulation of anabolic biomarkers (COL-II, SOX9 and OPG; *P* < 0.05).

**Conclusion:**

Extensive distraction (20 mm, ∼50% of original length) surpasses the physiological adaptive capacity of the knee joint, triggering irreversible degeneration via IL-1β/MMP-13-mediated sterile inflammation and OPG/RANKL-driven osteochondral uncoupling. While moderate distraction (10 mm, ∼25% length) remains compensatory, this pathological transition highlights the critical necessity of joint protection strategies when limb lengthening targets exceed ∼25% of the original bone length.

## Graphical Abstract

**Figure:**
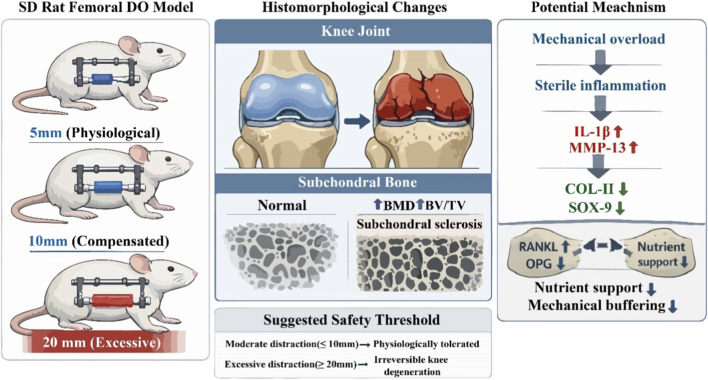

## Introduction

Distraction osteogenesis (DO) is widely regarded as the gold standard for treating large bone defects and limb deformities (Aktuglu et al., 2019; Bezstarosti et al., 2021; Seng and Oh, 2024). Relying on Ilizarov’s tension-stress principle, the technique uses gradual traction to stimulate tissue regeneration (Hosny, 2020). Yet, this regenerative process faces biological constraints. Unlike bone, which suggests high regenerative potential under tension, the surrounding soft tissue envelope—including muscles, fascia, and nerves—exhibits poor viscoelastic compliance and limited adaptive plasticity (Salimi et al., 2022). Consequently, as the limb lengthens, the soft tissues act as a restrictive tether. The resulting accumulation of passive tension is transmitted to adjacent joints, creating excessive joint reaction forces that may compromise joint integrity (Salimi et al., 2022).

The knee joint is particularly vulnerable to distraction-induced forces due to its complex weight-bearing anatomy. Complications such as stiffness, subluxation, and premature osteoarthritis are frequent in large-size limb lengthening (Bj Rge et al., 2021; Hosny, 2020). Despite established protocols for bone regeneration, the biological tolerance of articular cartilage remains a significant knowledge gap. Most studies have focused on qualitative outcomes rather than quantifying a specific safety threshold. Consequently, it remains unclear at what precise point physiological mechanical stimulation transitions into pathological degeneration, and which specific molecular mechanisms drive this deterioration.

Mechanobiological principles establish that chondrocyte function is dictated by the mechanical environment: physiological loads maintain homeostasis, whereas supraphysiological stress triggers catabolism Hodgkinson et al. (2022), Wang et al. (2023). Building on this, we hypothesize that the articular consequences of femoral distraction are governed by a specific length-dependent safety threshold. Mechanistically, we propose that soft tissue tension exceeding this threshold activates a sterile inflammatory response via the IL-1β/MMP-13 axis and uncouples the osteochondral unit through the OPG/RANKL pathway, accelerating the degeneration of the joint.

Therefore, this study investigates the dose-dependent impact of femoral DO on knee joint integrity using a graded distraction model (5, 10, and 20 mm) in SD rats. By integrating radiological, histological, and molecular analyses, we aim to uncover the specific mechanisms driving this transition. These insights will establish a theoretical framework for safe limb lengthening protocols and provide a biological rationale that may inform the development of future joint protection strategies in clinical settings.

## Materials and methods

### Animals

A total of 36 male Sprague-Dawley rats (SD, 8–10 weeks old, body weight 350 ± 20 g) were obtained from a licensed laboratory animal center. Animals were housed in groups of three per cage under specific pathogen-free conditions (22 °C ± 2 °C, 50%–60% humidity, 12 h light/dark cycle) with free access to standard chow and water. All experimental procedures were subjected to review and approval by the Ethics Committee of our institute (Approval number: IACUC-20241009-02). The study was conducted following the ethical standards for animal research in China.

### Surgical technique

Rats were anesthetized with an intraperitoneal injection of 2% pentobarbital sodium (40 mg/kg, Sigma, US) and placed in the lateral decubitus position. Prior to the osteotomy, the mean original length of the femur (from the greater trochanter to the lateral condyle) in these rats was measured using a digital caliper to be 40.52 ± 3.12 mm. This baseline measurement was used to calculate the relative percentage of limb lengthening for each experimental group. The right thigh was shaved and disinfected, and a longitudinal lateral incision was made over the mid-diaphysis of the femur. After blunt dissection of the muscles, a custom mini unilateral external fixator was applied using four stainless-steel half-pins (1.2 mm in diameter) inserted perpendicular to the femoral shaft, two proximally and two distally. A transverse osteotomy was then performed at the mid-diaphyseal level between the inner two pins using a low-speed oscillating saw under continuous saline irrigation to minimize thermal necrosis. Care was taken to avoid injury to the surrounding soft tissues and the distal femoral metaphysis and knee joint. The wound was irrigated, hemostasis achieved, and the soft tissues and skin were closed in layers. Postoperatively, rats were kept on a heating pad until recovery from anesthesia and then returned to their cages. Prophylactic antibiotics (e.g., cefazolin 20 mg/kg, intraperitoneal; Shanghai Baite Pharmaceutical Co. Ltd., China) were administered for 3 days, and analgesia was provided with buprenorphine (0.05 mg/kg, subcutaneous, twice daily for 3 days; Shanghai Baite Pharmaceutical Co. Ltd., China).

### Do protocol

Rats were randomly assigned to three groups (n = 12/group). Following a 5-day latency phase, distraction commenced at a rate of 0.5 mm/day (Graphical Abstract). The distraction magnitudes were selected to represent distinct clinical scenarios. A 5 mm distraction in the control group corresponds to a conservative lengthening of approximately 12% of the original bone length. Group A underwent a 10 mm distraction, representing about 25% of the original length. Group B received a 20 mm distraction, an extensive lengthening of roughly 50% designed as a maximal model to probe the biological limits of the knee joint and the mechanisms of irreversible failure. After completion of the distraction phase, the fixator was maintained in place during a consolidation phase until the predetermined endpoints. At the end of the consolidation phase, animals were euthanized by anesthesia overdose (2% pentobarbital sodium, 100 mg/kg, Sigma, US) for imaging and tissue collection.

### Bone formation outcomes *in Vivo*

Under brief anesthesia using isoflurane, a digital radiographic test using the HF400VA system (MIKASA, Tokyo, Japan) was performed every 2 weeks on the right femur of each rat to confirm the position of the fixation device, the quality of the regenerate, and the alignment of the limb. At 6 weeks of the consolidation phase, the collected femurs were fixed in a 4% paraformaldehyde solution for 48 h at room temperature for a micro-computed tomography (micro-CT) scan.

For three-dimensional assessment of the knee joint, the right distal femur, including the femoral condyles and adjacent metaphysis, was scanned using a high-resolution micro-CT system. Specimens (n = 5/group) were placed in a fixed orientation, and images were acquired at an isotropic voxel size of 9 μm, 80 kV, 200 µA for 240 ms (SkyScan, Bruker, Germany). Reconstruction was performed using the manufacturer’s software, and regions of interest (ROIs) were defined around the distal femoral epiphysis and subchondral bone plate. Quantitative analysis of callus formation within the ROIs included measurements such as Bone Mineral Density (BMD), Percentage of bone volume to total volume (BV/TV), Trabecular Thickness (Tb. Th), and Trabecular Separation (Tb. Sp). Morphological changes in the articular surface and joint space were qualitatively evaluated in multiplanar reconstructions.

### Histomorphometric evaluation

Following imaging, distal femoral specimens were fixed in 4% paraformaldehyde for 24–48 h and subjected to decalcification in 10% ethylenediaminetetraacetic acid (EDTA, pH 7.4) at room temperature with frequent solution changes. Complete decalcification was radiographically verified before specimens were dehydrated in a graded ethanol series, cleared in xylene, and embedded in paraffin. Serial sagittal sections (4–5 μm thick) were obtained through the femoral condyle using a rotary microtome (Leica RM2135, Germany). Serial sections were stained with hematoxylin and eosin (H&E, Servicebio G1004, China) to evaluate general morphology, Safranin O-Fast Green (Servicebio G1053, China) to assess proteoglycan content and cartilage matrix integrity, and Masson’s trichrome (Servicebio G1006, China) to visualize collagen deposition and subchondral bone remodeling. The standardized Osteoarthritis Research Society International (OARSI) scoring system for rats was employed to quantitatively assess the severity of articular cartilage degeneration (Gerwin et al., 2010). The scoring was performed on Safranin O-Fast Green stained sections, which were evaluated for cartilage matrix loss, chondrocyte integrity, and surface deformation. Each section received a score from 0 (normal) to 24 (complete bone-on-bone contact or denudation), calculated by multiplying the grade (0–6, representing lesion depth) by the stage (0–4, representing the horizontal extent of involvement).

For immunohistochemical analysis, paraffin sections were deparaffinized, rehydrated, and subjected to antigen retrieval in citrate buffer (pH 6.0) using a microwave oven. Endogenous peroxidase activity was blocked with 3% hydrogen peroxide, and non-specific binding was blocked with 5%–10% normal serum. Sections were incubated overnight at 4 °C with primary antibodies against interleukin-1β (IL-1β; Proteintech 26048-1-AP, 1: 100, China), matrix metalloproteinase-13 (MMP-13; Proteintech 18165-1-AP, 1: 100, China), type II collagen (COL-II; Huabio HA722733, 1: 200, China), SRY-box transcription factor 9 (SOX9; Huabio ET1611-56, 1: 200, China), osteoprotegerin (OPG; Proteintech 31766-1-AP, 1: 200, China), and receptor activator of nuclear factor-κB ligand (RANKL; Proteintech 66610-1-Ig, 1: 400, China) at appropriate dilutions. After washing, sections were incubated with horseradish peroxidase-conjugated secondary antibodies (ZSGB-BIO, China), developed with 3,3′-diaminobenzidine (DAB), and counterstained with hematoxylin. Images were captured from predefined regions of interest (ROIs) in the femoral condyle, and six random fields per ROI were analyzed under ×200 magnification using DP26 imaging software (OLYMPUS, Japan) in a blinded manner. The integrated optical density of the positively stained area or cells (brown), normalized to tissue area, was semi-quantified using Image Pro Plus 6.0 software.

### Statistical analysis

All quantitative data are presented as mean ± standard deviation. Normality of data distribution was assessed using the Shapiro-Wilk test, and homogeneity of variances was evaluated using Levene’s test. For normally distributed data with equal variances, comparisons among the three groups were performed using one-way analysis of variance (ANOVA) followed by Tukey’s *post hoc* test. For the primary molecular outcome (e.g., MMP-13 expression in Group B vs. Control), with an effect size (*d*) of 2.15 and a significance level (*α*) of 0.05, the total sample size of n = 5/group for IHC and micro-CT yielded a statistical power (1 - *β*) of 0.88. This exceeded the standard threshold of 0.80, confirming that the study was sufficiently powered to detect significant differences in molecular and radiological outcomes. For non-normally distributed data, the Kruskal-Wallis test followed by Dunn’s multiple comparison test was used. Statistical significance was *P* value <0.05. Statistical analyses were performed using GraphPad Prism software (version 10.0, US).

## Results

**FIGURE 1:**
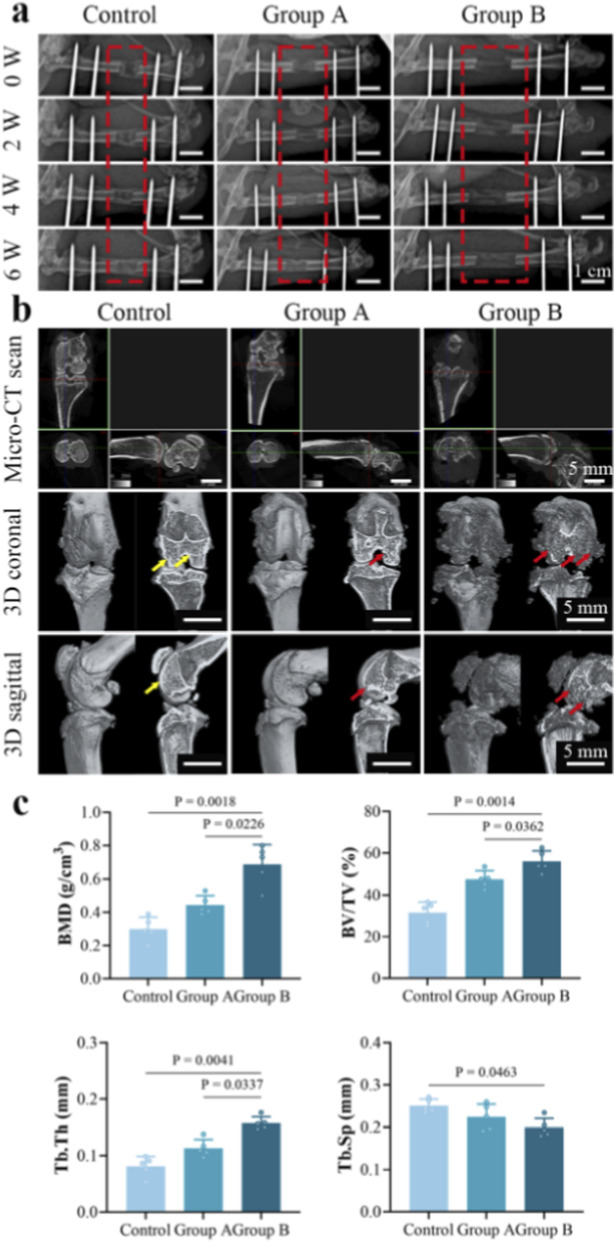
Radiographic evaluation of femoral distraction regeneration and Micro-CT assessment of knee joint morphology across different distraction lengths. **(a)** Serial X-rays of the distraction area at 2, 4, and 6 weeks of consolidation. **(b)** Micro-CT 3D reconstructions of the distal femur at 6 weeks. Group A showed a relatively preserved articular surface (yellow arrows) without evident osteophytes. Group B displayed significant degenerative changes, including surface irregularity, sclerosis, marginal osteophytes (red arrows), and joint narrowing, suggesting osteoarthritis caused by excessive tension. **(c)** Quantification of BMD, BV/TV, and Tb. Th in Group B were significantly elevated compared to the Control and Group A (n = 5/group).

Radiographic analysis demonstrated an inverse relationship between bone healing quality during femoral distraction osteogenesis and the magnitude of distraction (Figure 1a). The control group achieved optimal healing, with cortical continuity fully restored after 6 weeks of consolidation. While Group A underwent double the distraction length of the Control group, it still attained successful osseointegration, indicating that this extent of lengthening remains within the physiological compensatory capacity of the rat femur. In contrast, Group B exhibited clear signs of delayed or non-union. The excessive mechanical tension likely disrupted the local microenvironment necessary for callus formation, possibly through vessel over-stretching and subsequent ischemia.

Micro-CT assessment revealed that, unlike the smooth articular surface preserved in the Control group, the knee joints in Group B exhibited significant degenerative changes characteristic of osteoarthritis (Figure 1b). Joint space narrowing and osteophyte formation indicated that excessive mechanical stress led to cartilage attrition and aberrant subchondral bone remodeling. Quantitative analysis of the subchondral bone within the ROIs (Figure 1c) showed significantly higher values in Group B for BMD (0.68 ± 0.11 vs. 0.44 ± 0.05 vs. 0.3 ± 0.07 g/cm^3^, *P* < 0.05), BV/TV (55.94% ± 5.08% vs. 47.43% ± 4.03% vs. 31.55% ± 5.17%, *P* < 0.05), and Tb. Th (0.15 ± 0.01 vs. 0.11 ± 0.01 vs. 0.08 ± 0.01 mm, *P* < 0.05) compared to Group A and the Control group. Conversely, Tb. Sp was significantly lower in Group B than in the Control group (0.19 ± 0.02 vs. 0.25 ± 0.01 mm, *P* < 0.05). These findings suggest that distraction length exerted dose-dependent deleterious effects on the adjacent joints. The observed structural deterioration is consistent with a predicted increase in contact force across the articular surface. This mechanical environment likely originates from the passive tension of over-stretched soft tissues, including muscles and fascia.

**FIGURE 2:**
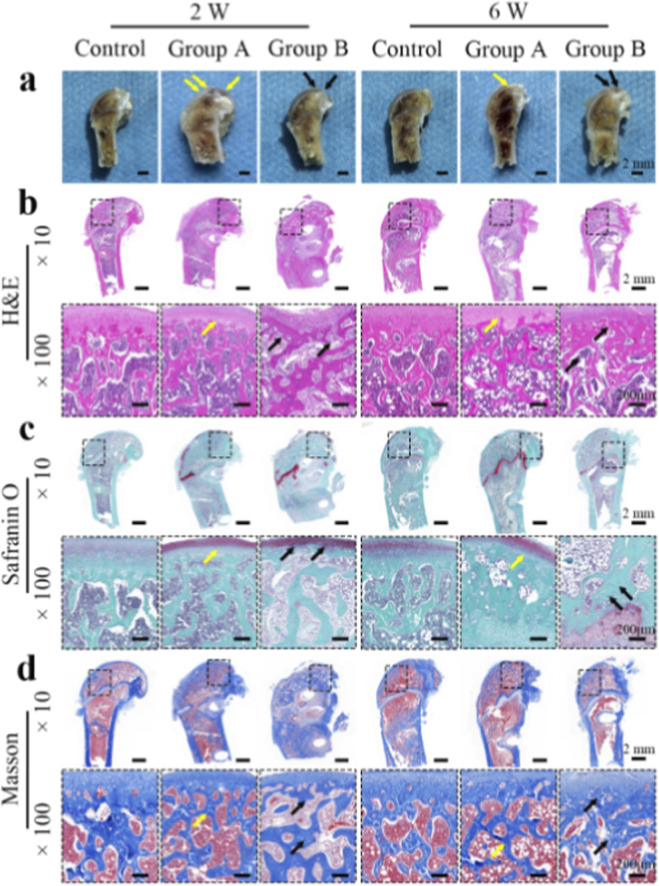
General images and histological staining of distal femoral cartilage. **(a)** Group A showed mild dullness and Group B exhibited progressive erosion and osteophytes (black arrows) by 6 weeks. **(b)** H&E: Group A showed mild clustering (yellow arrows) and Group B progressed from fibrillation (2 weeks) to deep fissures and necrosis (black arrows, 6 weeks). **(c)** Safranin O: Group A showed slight superficial loss (yellow arrows) and Group B demonstrated significant proteoglycan depletion (black arrows) by 6 weeks. **(d)** Masson’s Trichrome: Groups A and Group B exhibited disorganized fibers (yellow/black arrows) and subchondral sclerosis at 6 weeks. Controls remained histologically normal across all stains.

General imaging revealed a smooth, intact hyaline cartilage surface in the Control group throughout the consolidation phase (Figure 2a). Group A exhibited minimal alterations, indicating physiological tolerance to the distraction force. Group B, in contrast, displayed severe mechanical degeneration that progressed from surface dullness at 2 weeks to macroscopic erosion, cartilage thinning, and osteophyte formation by 6 weeks, a morphology consistent with advanced osteoarthritis. Besides, H&E staining showed that excessive mechanical stress in Group B disrupted the chondrocytes’ columnar arrangement (Figure 2b). Key pathological features, such as surface fibrillation and chondrocyte cloning, were all significantly more severe at 6 weeks than at 2 weeks. Safranin O staining revealed a marked reduction in red staining intensity in Group B, indicating severe proteoglycan depletion (Figure 2c). Since proteoglycans are essential for resisting compressive loads, their loss renders the cartilage highly susceptible to further mechanical damage. Quantitative OARSI scoring confirmed the dose-dependent severity of cartilage damage. After 6 weeks of consolidation, the OARSI scores in Group B (15.4 ± 3.05) were significantly higher than those in Group A (4.6 ± 1.81, *P* < 0.001) and the Control group (1.4 ± 0.54, *P* = 0.026). Masson’s Trichrome staining confirmed structural deterioration in Group B, the collagen network appeared disorganized and fibrotic, losing typical hyaline characteristics (Figure 2d and Supplementary Figure S1). Collectively, these results indicated that extensive femoral distraction (20 mm) induced severe, progressive degeneration of the adjacent articular cartilage. While moderate distraction (10 mm) induced only mild adaptive changes, 20 mm distraction exceeded the biological limit of the knee joint, triggering a pathological cascade indistinguishable from secondary osteoarthritis.

**FIGURE 3:**
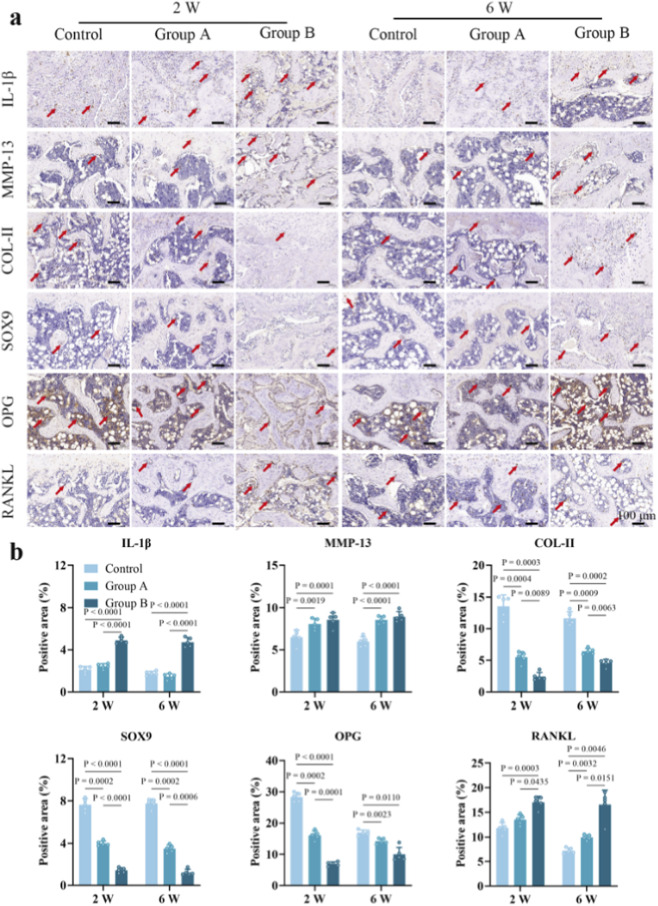
Representative immunohistochemical staining images and semi-quantitative analyses of the distal femoral cartilage sections. **(a,b)** IL-1β and MMP-13 expression (red arrows) significantly increased in Group B at 2 weeks and 6 weeks, primarily localized to superficial and middle chondrocytes. COL-II and SOX9 showed robust staining in Controls but progressive downregulation in Group B, signifying phenotypic loss. Group B further exhibited suppressed OPG and elevated RANKL at the subchondral interface, indicating a metabolic imbalance favoring osteoclastogenesis (n = 5/group).

At 2 weeks of consolidation phase, positive staining for inflammatory and osteoclast-related biomarkers in Group B was concentrated in the central region of the ROIs, unlike the biomarkers associated with cartilage formation (Figure 3a). Semi-quantitative analysis indicated that expression levels of IL-1β (Group B vs. Group A vs. Control: 4.89% ± 0.29% vs. 2.43% ± 0.23% vs. 2.10% ± 0.31%, *P* < 0.05), MMP-13 (8.56% ± 0.83% vs. 8.07% ± 0.65% vs. 6.48% ± 0.83%, *P* < 0.05), and RANKL (17.04% ± 1.22% vs. 13.48% ± 1.02% vs. 11.8% ± 1.13%, *P* < 0.05; Figure 3b) were elevated in Group B relative to both Group A and the Control. In contrast, expression of COL-II (2.44% ± 0.64% vs. 5.48% ± 0.87% vs. 13.55% ± 1.78%, *P* < 0.05), SOX9 (1.42% ± 0.23% vs. 3.99% ± 0.23% vs. 7.63% ± 0.59%, *P* < 0.05), and OPG (6.93% ± 0.64% vs. 16.14% ± 1.4% vs. 28.37% ± 1.42%, *P* < 0.05) was lower in Group B. By 6 weeks, IL-1β, MMP-13, and RANKL levels remained higher in Group B (4.71% ± 0.43%, 8.93% ± 0.61%, and 11.96% ± 0.7%) compared to Group A (1.45% ± 0.28%, 8.56% ± 0.43%, and 9.94% ± 0.51%; *P* < 0.05) and the Control (1.76% ± 0.19%, 5.96% ± 0.58%, and 7.23% ± 0.59%; *P* < 0.05). Similarly, expression of COL-II, SOX9, and OPG was lower in Group B (4.58% ± 0.56%, 1.22% ± 0.29%, and 19.42% ± 2.57%) than in Group A (6.42% ± 0.45%, 3.49% ± 0.37%, and 16.93% ± 0.84%; *P* < 0.05) and the Control (11.66% ± 1.04%, 7.76% ± 0.42%, and 11.43% ± 0.85%; *P* < 0.05). Together, the immunohistochemical analysis suggests that excessive distraction length may shift knee cartilage metabolism from an anabolic toward a catabolic and inflammatory state. This shift was evidenced by the downregulation of protective and synthetic markers (COL-II, SOX9, and OPG) alongside the upregulation of destructive mediators (IL-1β, MMP-13, and RANKL). These results suggest that the IL-1β/MMP-13 axis drives extracellular matrix degradation, while a dysregulated RANKL/OPG ratio mediates subchondral bone pathology, providing a pathological basis for the secondary osteoarthritis observed in Group B.

## Discussion

DO has been extensively validated as the more effective strategy for correcting large bone defects and limb discrepancies (Barakat et al., 2020; Calder et al., 2022; Seng and Oh, 2024). Previous research has largely concentrated on optimizing the rhythm and rate of distraction to enhance bone regeneration within the distraction gap (Hu et al., 2024). Although clinical reports have occasionally documented joint stiffness or subluxation after extensive lengthening, experimental data defining a specific safety threshold for distraction length with respect to articular cartilage integrity remain scarce (Bj Rge et al., 2021; Frost et al., 2023). This study bridges this gap by demonstrating that the deleterious effects on the knee joint are strictly length-dependent. We identified that while physiological distraction (up to 10 mm) is well-tolerated, excessive distraction (20 mm) represents a magnitude where biological failure becomes evident, suggesting that the joint’s tolerance is overwhelmed between the 25% (10 mm) and 50% (20 mm) lengthening marks.

The mechanical mechanism warrants careful interpretation in the context of Ilizarov’s Law of Tension-Stress, which posits that gradual traction stimulates tissue regeneration (Hu et al., 2024). While numerous studies have confirmed that this principle promotes osteogenesis and angiogenesis in the distraction area, our results suggest a paradoxical effect on the adjacent joint (Frommer et al., 2022; Yang et al., 2022). Previous research has documented that extensive distraction leads to muscle fibrosis and contracture due to the exhaustion of muscle adaptability (Salimi et al., 2022). In this study, while our results clearly demonstrate a length-dependent degenerative response, it is important to note that the increased joint reaction force might be inferred from the resulting histopathological changes. The severe cartilage degeneration observed in Group B signifies that the mechanical stress exceeded the physiological compliance of the joint, highlighting the critical value of considering the muscle-joint axis rather than focusing solely on the bone in large distraction length.

Existing literature on cartilage mechanobiology has established that cyclic mechanical overload triggers a pro-inflammatory response in chondrocytes (Cai et al., 2024; Hikida et al., 2025). Consistent with these established osteoarthritis models, our immunohistochemical analysis revealed a significant upregulation of IL-1β and MMP-13 in the 20 mm distraction group. This confirms that the continuous static tension from limb lengthening creates a local environment of sterile inflammation similar to impact injuries. The value of this observation lies in confirming that distraction-induced joint damage is an active, biologically mediated enzymatic degradation process driven by the IL-1β/MMP-13 axis, rather than simple physical wear and tear.

It is well recognized in orthopaedic research that physiological mechanical loading is essential for maintaining cartilage nutrition and chondrocyte phenotype (Culliton and Speirs, 2021; Wang et al., 2023). Indeed, our Group A (10 mm) results support this, showing preserved COL-II and SOX9 expression, likely representing an adaptive response to moderate tension. However, in stark contrast, existing studies indicate that supra-physiological strain can induce chondrocyte dedifferentiation (Lohberger et al., 2025; Sobue et al., 2019; Wang et al., 2023). Our data from Group B strongly corroborate this, demonstrating a widespread loss of SOX9 and COL-II. This study, therefore, provides crucial *in vivo* evidence delineating the boundary between anabolic stimulation and catabolic suppression. These results indicate that beyond a 10 mm lengthening, the metabolic balance capacity of the adjacent joint cartilage declines irreversibly, resulting in its degradation.

Furthermore, contemporary osteoarthritis research has shifted focus from the cartilage surface alone to the integrated osteochondral unit, suggesting that subchondral bone remodeling plays a pivotal role in disease progression (Hu et al., 2021; Liang et al., 2024). In this study, the Micro-CT and IHC results (specifically the dysregulated OPG/RANKL ratio) revealed distinct subchondral sclerosis in the 20 mm group. Unlike previous studies that isolated cartilage changes, our work suggests that distraction-induced forces disrupt the crosstalk between the articular cartilage and the underlying bone. This insight is valuable as it suggests that therapeutic interventions for distraction-induced arthropathy must target both the cartilage and the subchondral bone remodeling to be effective.

Current clinical protocols for managing joint complications, such as prophylactic joint fixation or intensive physiotherapy, often rely on empirical experience (Liu et al., 2021; Liu et al., 2023; Pawson et al., 2024). Patients undergoing greater limb lengthening face a higher risk of complications due to increased EFT and BUT (Liu et al., 2021; Liu et al., 2023). The biological relevance of our model is supported by the relative lengthening percentages. With a mean rat femoral length of 40.52 mm, our Group A (10 mm, ∼25%) represents the upper limit of clinically acceptable lengthening, whereas Group B (20 mm, ∼50%) models extreme scenarios where catastrophic joint degeneration is common. This distinction clarifies the physiological tolerance in Group A and the severe pathological cascade initiated in Group B. Consequently, our findings establish a theoretical framework that underscores the need for close monitoring of joint health during extensive lengthening. These results support the hypothesis that preventive measures, such as soft tissue release, could mitigate tension-induced damage, a premise that warrants further investigation in clinical cohorts.

Several limitations should be acknowledged. First, the total sample size was 36 rats, but only a subset (n = 5 per group) was used for micro-CT quantification. A larger cohort would be required to elucidate more nuanced molecular shifts during the early distraction phase. Second, the mechanical loading pattern of the rat knee differs from that of humans, which may influence the translatability of the threshold values. Finally, while we inferred increased joint reaction force from morphological changes, direct measurement of intra-articular pressure was not performed. Future research incorporating dynamic assessments, such as gait analysis, force plate measurements, or the use of intra-articular pressure sensors, is warranted to quantify the exact mechanical threshold during the distraction and consolidation phases.

## Conclusions

This study demonstrates that while a 10 mm femoral distraction (representing approximately 25% of the native bone length) is physiologically tolerated in rats, an extensive 20 mm distraction (50% lengthening) precipitates irreversible knee joint degeneration driven by soft tissue tension overload. This supraphysiological mechanical stress accelerates the deterioration of the osteochondral unit by activating the IL-1β/MMP-13 catabolic axis and dysregulating OPG/RANKL signaling within the subchondral bone. While these findings in a rat model offer hypothesis-generating insights into the risks associated with massive limb lengthening (∼25% of the original bone length), they underscore the potential necessity of proactive joint protection strategies. Further clinical investigations are essential to determine whether these biological and pathological mechanisms translate effectively to clinical practice.

## Data Availability

The original contributions presented in the study are included in the article/Supplementary Material, further inquiries can be directed to the corresponding authors.
