# Supplementary figures and images for: Effect of distraction length on the morphology of knee cartilage in a rat model of femoral distraction osteogenesis
Source: Front Physiol. 2026 Mar 16;17:1779440. doi: 10.3389/fphys.2026.1779440 (PMC13033532; doi:10.3389/fphys.2026.1779440)

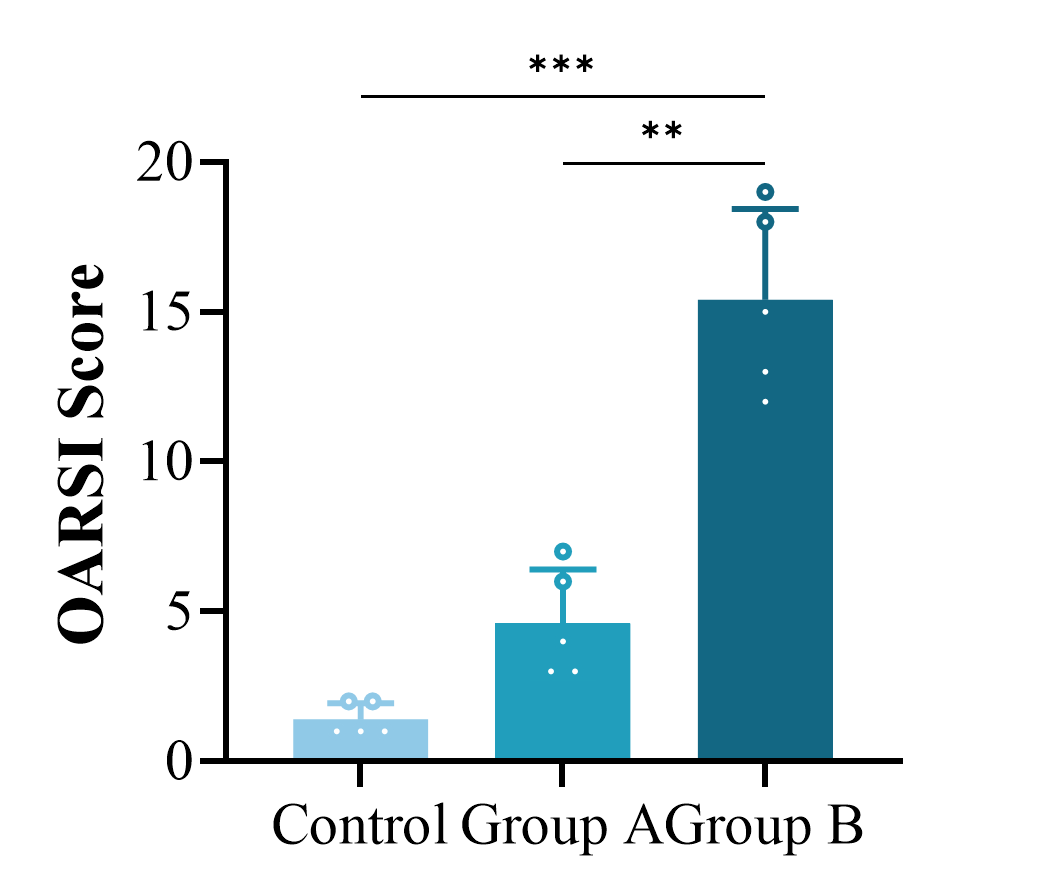

Supplement: Supplementary file 1 [file Image1.tif]
